# Peste des petits ruminants in Africa: a review of currently available molecular epidemiological data, 2020

**DOI:** 10.1007/s00705-020-04732-1

**Published:** 2020-07-11

**Authors:** William G. Dundon, Adama Diallo, Giovanni Cattoli

**Affiliations:** 1 Animal Production and Health Laboratory, Animal Production and Health Section, Joint FAO/IAEA Division, Department of Nuclear Sciences and Applications, International Atomic Energy Agency, Friedenstrasse, 1, 2444 Vienna, Seibersdorf Austria; 2 CIRAD, UMR ASTRE, ISRA/LNERV, Dakar-Hann, Senegal; 3 UMR ASTRE, Univ. Montpellier, 34398 Montpellier, France; 4 CIRAD, INRA, 34398 Montpellier, France

## Abstract

Small ruminants (e.g., sheep and goats) contribute considerably to the cash income and nutrition of small farmers in most countries in Africa and Asia. Their husbandry is threatened by the highly infectious transboundary viral disease peste des petits ruminants (PPR) caused by peste-des-petits-ruminants virus (PPRV). Given its social and economic impact, PPR is presently being targeted by international organizations for global eradication by 2030. Since its first description in Côte d’Ivoire in 1942, and particularly over the last 10 years, a large amount of molecular epidemiological data on the virus have been generated in Africa. This review aims to consolidate these data in order to have a clearer picture of the current PPR situation in Africa, which will, in turn, assist authorities in global eradication attempts.

**Electronic supplementary material:**

The online version of this article (10.1007/s00705-020-04732-1) contains supplementary material, which is available to authorized users.

## Introduction

Peste des petits ruminants (PPR), a highly contagious viral disease of small ruminants, continues to cause the death of millions of sheep and goats annually and is a constant threat to the livelihoods of subsistence farmers in many countries in Africa, the Middle East, and Asia.

The disease is caused by peste-des-petits-ruminants virus (PPRV) of the family *Paramyxoviridae*, subfamily *Orthoparamyxovirinae*, genus *Morbillivirus*, species *Small ruminant morbillivirus* [1]. The genome encodes two non-structural proteins, C and V, and six structural proteins arranged in the order nucleoprotein (N), phosphoprotein (P), matrix protein (M), fusion protein (F), hemagglutinin (H) and viral RNA-dependent RNA polymerase (L) [2, 3]. The first sequence from a PPRV isolate was generated by Diallo et al. in 1994 [4] when they cloned the N gene of a vaccine strain originating from Nigeria in 1975, which was followed by the sequencing of the F protein gene [5]. The sequencing of the N and F genes also allowed the development of important molecular diagnostic tools for PPRV that are routinely used today by many [6–10].

Even though the virus is serologically monotypic, PPRV strains have been classified into four genetically distinct lineages (I, II, III and IV) based on partial sequences of the N and F genes [11–13]. A recent study has shown that inter-lineage resolution is better when N gene sequences are used [14], compared to the H or F gene. The first PPRVs identified in Africa belong to lineages I, II and III, while viruses belonging to lineage IV have been found primarily in Asia and the Middle East [12, 13]. Since 2008, however, lineage IV viruses have also been regularly reported in different African countries, and this lineage is becoming the predominant lineage on the continent [13, 15–17].

Africa consists of 54 sovereign states covering an area of 30.3 million km^2^ and has an estimated human population of just over 1.2 billion. According to FAO there were close to 423 million goats and 381 million sheep in Africa in 2014 [18]. The impact of PPR on this small ruminant population is therefore huge and has enormous implications for Africa.

Up to June 2020, there have been no official reports on PPR in the following southern African countries: Botswana, Eswatini, Lesotho, Malawi, Mozambique, Namibia, South Africa, Zambia, and Zimbabwe. In addition, PPR has never been reported on the Atlantic Ocean island nations of Cape Verde, St Helena, and São Tomé and Principé or the Indian Ocean islands of Madagascar, Mauritius and Seychelles. However, all of these islands are considered to be at risk of PPR introduction, as they import live animals from mainland Africa.

One of the mandates of the Animal Production and Health Laboratory (APHL) of the Joint FAO/IAEA division is to assist member states in the diagnosis and control of transboundary animal diseases in their countries, including PPR. This involves the analysis of, and virus isolation from, pathological samples collected by member states and sent to the APHL through the biosafety-3-level facilities of the Austrian Agency for Health and Food Safety (AGES). This has provided APHL with very relevant data on PPR, some not yet published and now shared in this review.

Building on the successful global eradication of rinderpest virus, the Food and Agricultural Organization of the United Nations (FAO) and the World Animal Health Organization (OIE) have selected PPR for global eradication by 2030 [19]. This will be supported by the availability of a number of efficacious live-attenuated vaccines (e.g., Nigeria 71/1, Sungri 96, Arasur 87 and Coimbatore 97) [20–22]. Molecular epidemiology provides important information on the transboundary movement of viruses such as PPRV. In fact, molecular epidemiological investigations satisfy one of the recommendations of the global Strategy for the Control and Eradication of PPR which states that each country needs to define and implement robust monitoring plans to gain a good understanding of the circulation (or non-circulation) of PPRV in their country.

**Fig. 1:**
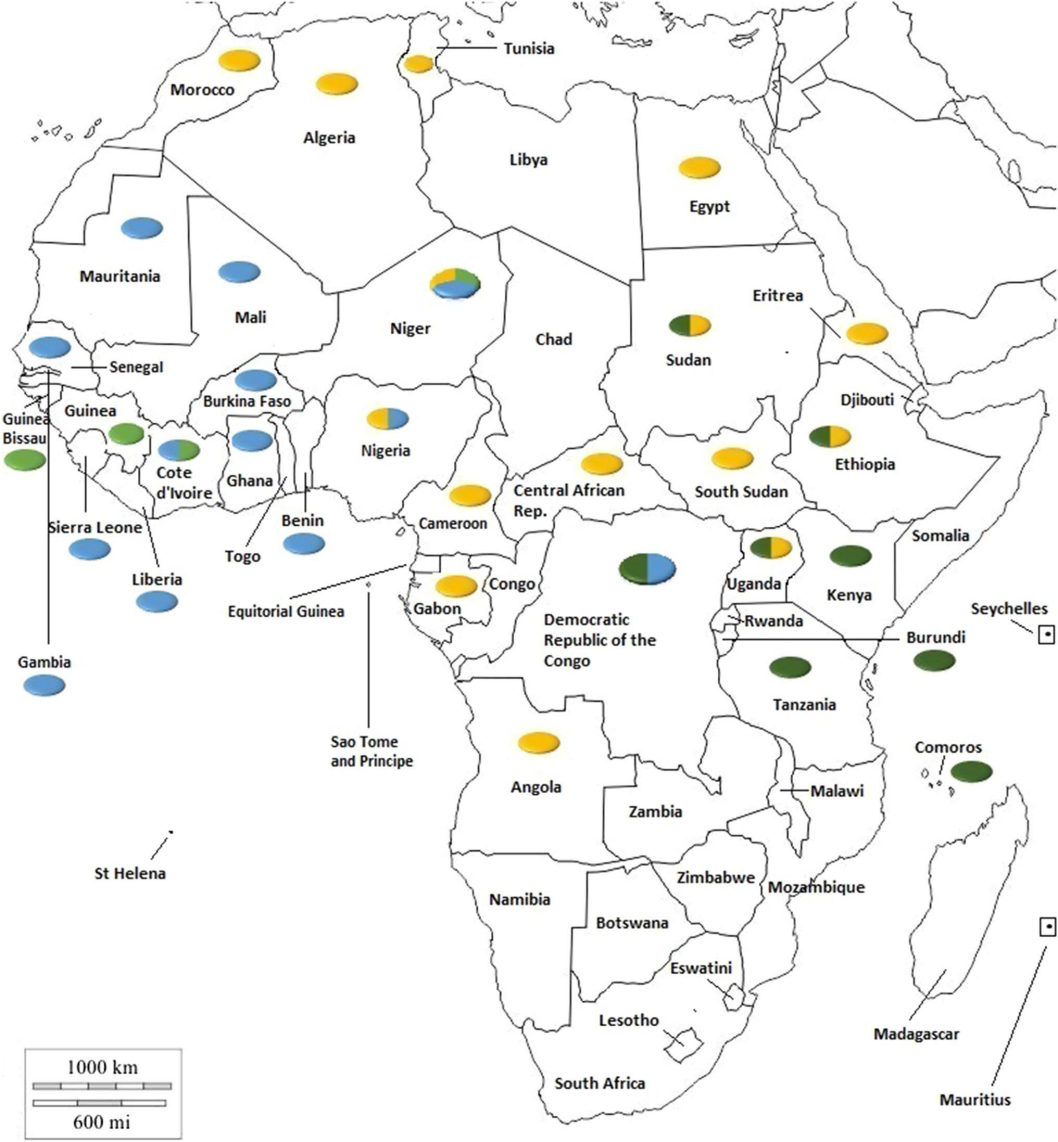
Distribution of PPRV lineages in Africa. Colours in the circles indicate the viral lineages reported in the country: lineage 1, light green; lineage II, blue, lineage III, dark green; lineage IV, yellow. Adapted with permission from www.d-maps.com

The aim of this review is, therefore, to provide updated molecular epidemiological data on each of the African countries from which data are currently available (June 2020). (For convenience, the data are also summarized in Fig. 1).

## Algeria

**Fig. 2:**
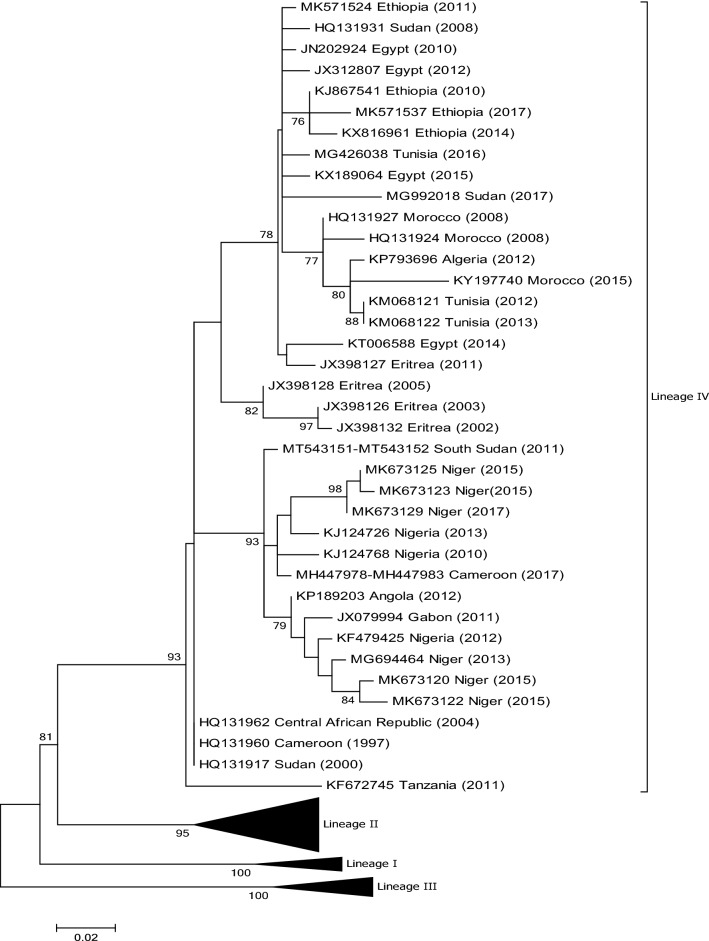
Phylogenetic tree based on partial sequences of the N protein (217 bp) gene of representative PPRV isolates of lineage IV. The tree was constructed using the maximum-likelihood (ML) method available in MEGA6, employing the Kimura 2-parameter model of nucleotide substitution and 500 bootstrap replications. The model was selected by MEGA6 as the best for the sequences being analysed

In February 2012, blood and oculo-nasal swab samples were collected from sheep and goats during an outbreak in central Algeria [23]. An analysis of the samples showed that the viruses belonged to lineage IV and clustered with PPRVs from Morocco and Tunisia (Fig. 2). The full genome sequence of an Algerian isolate collected in 2015 is available [24].

## Angola

In 2012, the APHL received samples from an outbreak of PPR reported in Angola [25]. Partial N and F gene sequence analysis (GenBank nos. KP189203 and KP189204) revealed that the causative virus belonged to PPRV lineage IV (Fig. 2).

## Burundi

**Fig. 3:**
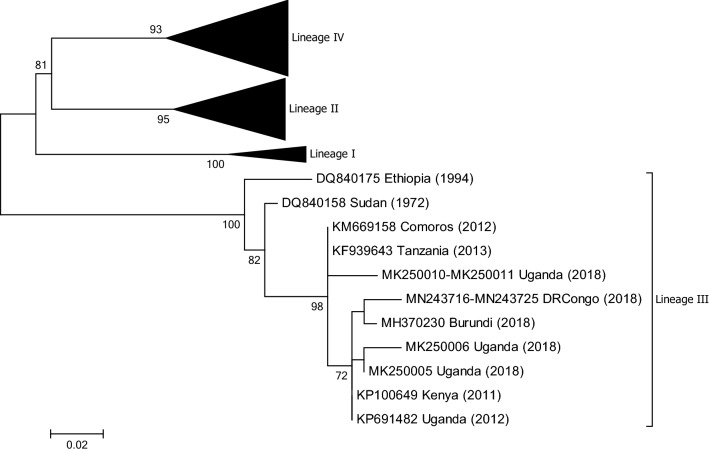
Phylogenetic tree based on partial sequences of the N protein (217 bp) gene of from representative PPRV isolates of lineage III. The tree was constructed using the maximum-likelihood (ML) method available in MEGA6, employing the Kimura 2-parameter model of nucleotide substitution and 500 bootstrap replications. The model was selected by MEGA6 as the best for the sequences being analysed

In December 2017, PPR emerged in Burundi, causing severe disease and killing more than 4,000 goats [26]. This was the first report of PPR in the country. Samples tested by conventional RT-PCR indicated the presence of PPRV, and phylogenetic analysis showed that the virus belonged to lineage III and showed a close relationship to PPRVs from Kenya, Uganda, and the Democratic Republic of Congo (DRC) in 2012 (Fig. 3). Interestingly, the outbreaks in Burundi coincided with the introduction of Boer goats (a breed of goat developed in South Africa for meat production) from Uganda through a project aiming at improving local goat production. During the implementation of the project, 2,200 goats were purchased in the Bushenyi and Mbarara regions of Uganda, transported by truck through Tanzania, and then distributed to farmers between 7 and 9, December 2017. The first cases of PPR appeared 12 days after the introduction of the new animals. A full genome sequence of an isolated virus (B3) has been deposited in GenBank (no. MK686066).

## Benin

**Fig. 4:**
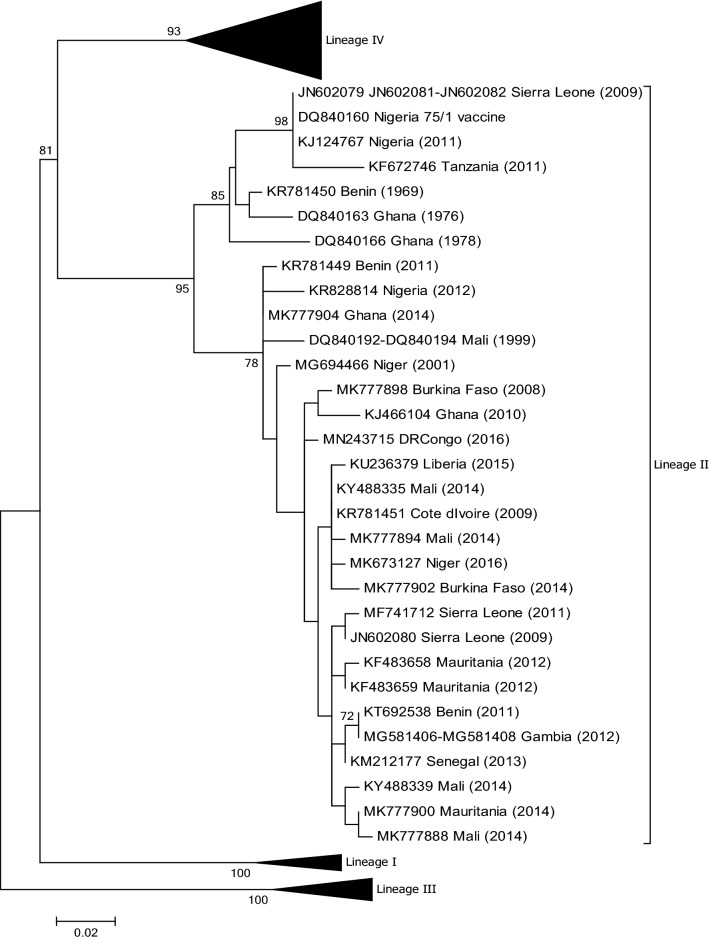
Phylogenetic tree based on partial sequences of the N protein (217 bp) gene of representative PPRV isolates of lineage II. The tree was constructed using the maximum-likelihood (ML) method available in MEGA6, employing the Kimura 2-parameter model of nucleotide substitution and 500 bootstrap replications. The model was selected by MEGA6 as the best for the sequences being analysed

PPR has been present in Benin since the early 1940s. Although PPR was officially named peste des petits ruminants by Gargadennec and Lalanne in Côte d’Ivoire in 1942, a similar syndrome was described in Dahomey (the former name of Benin) during the same period and was referred to as ‘*peste des espèces ovine et caprine’* (plague of ovine and caprine species) [27]. The presence of PPR has been regularly reported to the OIE by the Benin veterinary services [28]. Pathological and swab samples were collected from sheep and goats during disease outbreaks in 2011, and PPRV was isolated from positive samples in cell culture [27]. Phylogenetic analysis using partial N gene sequences showed that all of the isolates clustered within viral lineage II but fell into two distinct subclades (Fig. 4). In the same study, the full genome sequence of one of the isolates from 2011 (GenBank no. KR781449) was compared to the full genome sequence (GenBank no. KR781450) of a PPRV isolated from the lymph node of an infected goat in 1969 in Benin. A molecular clock analysis of complete PPRV genome sequences revealed that the lineage II viruses sampled arose in the early 1960s and that these viruses have most likely persisted in Benin since then [29].

## Burkina Faso

**Fig. 5:**
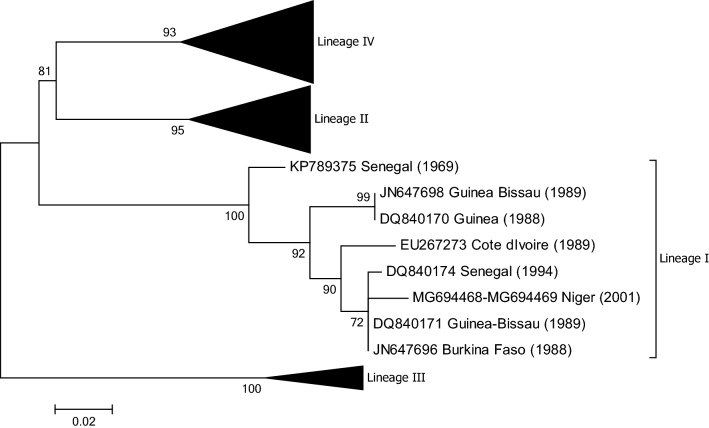
Phylogenetic tree based on partial sequences of the N protein (217 bp) gene of representative PPRV isolates of lineage I. The tree was constructed using the maximum-likelihood (ML) method available in MEGA6, employing the Kimura 2-parameter model of nucleotide substitution and 500 bootstrap replications. The model was selected by MEGA6 as the best for the sequences being analysed

According to Banyard et al. [12], regional reference laboratories confirmed the presence of PPR in Burkina Faso in 2008, although disease outbreak details are not available. A full sequence of the N gene (GenBank no. JN647696) of a virus collected in 1988 (Burkina 1988/1) which belongs to lineage I is available (Fig. 5). Burkina 1988/1 is one of three PPRVs that were isolated on lamb lung primary cells from pathological samples received at the Institut d'Elevage et de Médecine Vétérinaire des Pays Tropicaux (IEMVT), Maisons-Alfort, France, in September 1988 (Diallo A., unpublished data). The samples were collected from an animal suffering from respiratory distress during an outbreak affecting goats in the centre of Burkina Faso. In addition, the N gene sequences of PPRV RNA isolated from samples provided to the APHL that were collected in Burkina Faso in 2007 and 2009 have been determined (GenBank nos. MN564947-MN564948). Phylogenetic analysis showed that these viruses belong to lineage II (Fig. 4). Tounkara et al. [30] have recently analysed five partial N gene sequences generated from samples collected from goats and sheep in Burkina Faso in 2008 and 2014. All of the sequences belonged to viruses of lineage II.

## Cameroon

PPR has been reported in Cameroon, but detailed studies are limited [12, 31, 32]. There are seven GenBank submissions (GenBank nos. HQ131960, MH447978 to MH447983) of partial N gene sequences of viruses identified in goats in 1997 (n = 1) [12] and 2017 (n = 6). Phylogenetic analysis shows that they all belong to lineage IV (Fig. 2).

## Chad

There is a report of PPR in Chad dating back to 1995 that covers the period 1993 to 1994 [33]. A serological prevalence of 34% was recorded by ELISA following the testing of 475 sera. More recently, 3,546 sera collected from unvaccinated goats and sheep in 19 of the 23 regions in Chad were tested for antibodies to PPRV [34]. The overall seroprevalence was calculated as almost 53%. Despite these studies, there is no genetic information of PPRVs circulating in the country.

## Central African Republic

There is a single partial N gene sequence submission (GenBank no. HQ131962) from the Central African Republic obtained from a goat sample in 2004. The virus belongs to lineage IV (Fig. 2). No further information on PPR in the country is currently available.

## Comoros archipelago

Lineage III PPRVs were identified following an outbreak of PPR in 2012 [35] (Fig. 3). An overall PPRV antibody prevalence of 2.24% in indigenous domesticated ruminants was also reported by the authors.

## Côte d’Ivoire

The first-ever report of PPR globally was from Côte d’Ivoire in 1942 [36]. Since then, there have been relatively few published studies on PPR in the country. One of the first full genome sequences generated from a PPRV isolate was from a lineage I virus isolated in Côte d’Ivoire in 1989 (GenBank no. EU267273) [37] (Fig. 5). A second full genome sequence of a lineage II viral isolate recovered from lung samples of a goat collected in July 2009 has also been generated  by APHL (GenBank no. KR781451) (Fig. 4).

## Democratic Republic of Congo

In 2012 it was reported that FAO was assisting veterinary authorities in the DRC, as thousands of goats had been affected by PPR, of which 75,000 had already died from the disease [38]. More recently, the analysis of viral RNA from eleven goat samples collected in three provinces of the DRC in 2016 and 2018 identified viruses from two lineages, II and III (GenBank nos. MN243715 to MN243725) [39] (Figs. 3 and 4).

## Djibouti

Serum samples (n = 1,516) were tested using a competitive enzyme-linked immunosorbent assay (cELISA) by Teshale et al. [40]. Of these samples, 91 were positive, with an overall 6% prevalence of antibodies to PPRV, which is low compared to other serological studies. To date, there are no molecular epidemiological data available on PPRVs from Djibouti.

## Egypt

An outbreak of PPR in goats in Egypt was reported in 1990 [41]. Other outbreaks reports followed in 1993 [42] and 2010 [43], but it was not until 2014 that a virus, collected in 2010, was identified as belonging to lineage IV [44] (Fig. 2). There are more recent submissions in GenBank of sequences from lineage IV Egyptian viruses collected in 2012, 2014 and 2015. All of the viruses are closely related to viruses from Eritrea, Ethiopia, and Sudan and to viruses identified in Tunisia in 2016 (Fig. 2).

## Equatorial Guinea

There is no information on the presence of PPR in Equatorial Guinea available in the literature, and no reports have been submitted to the OIE.

## Ethiopia

The first published account of PPR in Ethiopia is from 1994 and described an outbreak in goats in the capital city, Addis Ababa [45]. PPRV was isolated from this outbreak at IEMVT, Maisons-Alfort, France, and shown to belong to lineage III [13]. The full genome of this virus was then sequenced in 2014 [46]. In 2016, the full genome of another PPRV isolate from the intestine of a goat suffering from severe clinical disease during an outbreak in 2010 was characterised and shown to belong to lineage IV [47], indicating that viruses from two lineages have been present at different times in the country (Figs. 2 and 3). However, two more recent studies on samples from Ethiopia from 2011, 2014 and 2017 only detected lineage IV PPRVs, suggesting that the lineage III viruses may have been replaced by lineage IV viruses [48, 49]. Currently, there are five full genome sequences of PPRVs from Ethiopia available in GenBank (nos. KJ867540, KJ867541, MK991798, MK991799 and MK991800).

## Eritrea

A paper published in 1998 by Sumption et al. [50] reported the detection of PPRV antigen by an immunofluorescent antibody test from ocular swabs collected from goats during an outbreak of PPR in Eritrea. Cosseddu et al. [15] analysed 41 samples collected from sheep and goats in 2002, 2003, 2005 and 2011, 34 of which were shown to be positive for PPRV by RT-PCR. The partial N gene sequences of seven of the samples were determined, and phylogenetic analysis identified them as belonging to lineage IV (Fig. 2).

## Gabon

According to Banyard et al. [12], there was serological evidence for the presence of PPRV in Gabon in 2007. The first molecular characterization of PPRV in the country was described following a PPR outbreak in sheep and goats in the southeast of the country in October 2011 [16]. Phylogenetic analysis of a partial N gene sequence showed that the virus belonged to lineage IV, and the authors concluded that it was more closely related to viruses circulating in neighbouring Cameroon. However, it appears that the virus is more closely related to PPRV isolates from Angola and Niger rather than Cameroon (Fig. 2).

## Gambia

As part of a rinderpest vaccination study, serum samples collected from sheep and goats between 1988 and 1989 were tested for the presence of antibodies to PPRV and shown to be positive [51]. Three pathological samples that had been collected from three goats suspected of being infected with PPR were sent to APHL in 2012 for further characterization. Sequencing of a fragment of the N (GenBank nos. MG581406 to MG581408) and F gene (GenBank nos. MG581409 to MG581411) and phylogenetic analysis showed that the viral RNA belonged to lineage II and showed a high level of sequence similarity to viruses from neighbouring countries (Fig. 4).

## Ghana

In the GenBank database, there are three partial N gene sequences from Ghana collected in 1976 (GenBank no. DQ840163) and 1978 (GenBank no. DQ840166 and DQ840167), respectively, that are associated with a study by Kwiatek et al. [52]. There is also a partial F gene sequence available (GenBank no. FR668075). These viruses belong to lineage II (Fig. 4), but in all four cases there is no epidemiological or outbreak information provided with the sequence data.

In 2010, pathological samples collected from sheep and goats suspected of being infected with PPRV in Ghana between September 2009 and March 2010 were analysed and shown to contain PPRV that belonged to lineage II [53] (Fig. 4). The full genome of one of the isolates obtained from the lung of a female sheep was sequenced (GenBank no. KJ466104). A recent report confirmed the presence of lineage II PPRV in Ghana in 2014 [30].

## Guinea

There are two partial N and F gene sequences available in the GenBank database, from 1988 and 1991, respectively (GenBank no. DQ840170 and FR667554), that indicate that the virus in Guinea in the late 1980s belonged to lineage I (Fig. 5). No additional epidemiological information associated with these sequences or updates on the PPR situation in Guinea are available.

## Guinea-Bissau

There are four nucleotide sequences available from Guinea-Bisseau from 1988 to 1991 in GenBank, two of which are partial N gene sequences from 1989 (no. JN647698 and DQ840171). No epidemiological data are associated with them. Phylogenetic analysis shows that the sequences are derived from lineage I isolates (Fig. 5).

## Kenya

PPR was confirmed serologically in Kenya in 1995, but the presence of the disease in the country was not declared to the OIE until 2007 [54, 55]. From 2006 to 2008, the virus spread rapidly and it is estimated that during this period over 2.5 million animals died as a result of PPR [55]. In May 2011 in north-western Kenya, tissue samples were collected from goats suspected of having died from PPR. RT-PCR-positive samples were sequenced and identified as belonging to PPRV lineage III (Fig. 3) [56].

## Liberia

The first characterization of PPRV in Liberia was reported in April 2015 following significant mortality in sheep and goats in the country [57]. The outbreaks were in the north-central part of Liberia, close to the border with Guinea. The full genome sequence of one isolate was determined (GenBank no. KU236379), and phylogenetic analysis revealed that the virus belonged to lineage II (Fig. 4).

## Libya

There are two seroprevalence studies on PPR in Libya. Both studies were undertaken in 2013 and confirmed the presence of the disease in the country, with a high overall sero-prevalence of between 33 and 59% [58, 59]. However, despite these studies, no genetic information on the circulating PPRV in Libya is currently available.

## Mali

A serological study undertaken to determine the seropositivity of 54 flocks of small ruminants for rinderpest in 1996 revealed that a high percentage (74%) of the flocks were positive for antibodies against PPRV [60]. A more recent study published in 2013 stated that 14 outbreaks of PPR had been officially reported by the national veterinary service in Mali between 2007 and 2011 [61]. This study also revealed a positivity of 43% among sheep and goats in the country. There are eleven gene sequences from Mali available in the GenBank database: three from 1999 (GenBank nos. DQ840192 to DQ840194) and eight from 2014 (GenBank nos. KY488320 to KY488322, KY488325, KY488335 to KY488337, and KY488339) Again, there is no epidemiological information associated with these gene sequences, although phylogenetic analysis reveals that they all belong to lineage II (Fig. 4). A more recent study included the analysis of eleven samples from goats in Mali [30]. One of the samples was collected in 1999, while the remaining 10 were ocular swabs collected in 2014. Phylogenetic analysis of partial N gene sequences from these samples indicated that all of the viruses belong to lineage II but indicated a clear evolution of the viruses over time (Fig. 4).

## Mauritania

Up until relatively recently, there were only a few reports on PPR inMauritania, primarily on disease description and serology [62, 63]. However, between January and March 2012, samples from three suspected outbreaks of PPR in Mauritania were collected and characterized in detail [64]. A phylogenetic analysis of N gene sequences showed that the virus circulating in Mauritania belonged to lineage II, similar to viruses in Senegal and Mali, but not Morocco or Algeria, where lineage IV viruses were circulating (Fig. 4). This highlighted the results of livestock movement from Mauritania to countries to the south. This observation was recently confirmed by Tounkara et al. [30] (GenBank no. MK777900; Fig. 2).

## Morocco

The first reported outbreaks of PPR in Morocco were in 2008. Thirty-six samples from sheep were collected and analysed, and a lineage IV PPRV was identified [13]. The complete genome sequence of a similar virus isolated from an alpine goat was published in 2013 [65]. Three successive vaccination campaigns during 2008–2011 and border surveillance resulted in the elimination of the virus from the country. However, in 2015, fresh outbreaks were reported. Analysis of the 2015 virus revealed that it also belonged to lineage IV. Figure 2 shows that viruses from Morocco are closely related to viruses from Algeria and Tunisia, suggesting transboundary movement between countries [66].

## Niger

Serological investigations carried out by various authors confirmed the presence of PPR in Niger from the 1990s onwards [67, 68]. The first genetic characterization of viruses circulating in the country was carried out by Tounkara et al. [69]. Samples taken from goats during PPR outbreaks in 2001 and 2013 were analysed, and viruses from lineage I, II and IV were identified (Figs. 2, 4 and 5). More recent samples were collected from goats and sheep at locations throughout Niger between 2011 and 2017 [70]. Twelve PPRV-positive samples were characterized by partial sequencing of the N gene (GenBank nos. MK673120 to MK673131), and the viruses identified were from lineages II and IV only [70]. The most recent sequences differed significantly from those reported in 2001 and 2013 by Tounkara et al. [69], highlighting the continuing evolution of PPRV in Niger. Two separate subclades of the lineage IV viruses were identified. In one of them, the sequences clustered with Nigerian sequences that have previously been assigned to subclade IV-NigA by Woma et al. [17]. The sequences of the remaining samples clustered with sequences from Nigeria that had been previously been assigned to subclade IV-NigB [17] (Fig. 2).

## Nigeria

There are a number of studies describing PPR outbreaks in Nigeria in the 1970s, 1980s and 1990s [71–74]. However, it was only in 1975 and 1976 that PPRV from the country was isolated for the first time [75]. The current live-attenuated PPRV vaccine was derived from one of those isolates, Nigeria 75/1 [76] (Fig. 4). The full genome sequence of another lineage II PPRV isolate from Nigeria in 1976 was generated by Chard et al. in 2008 [37].

**Fig. 6:**
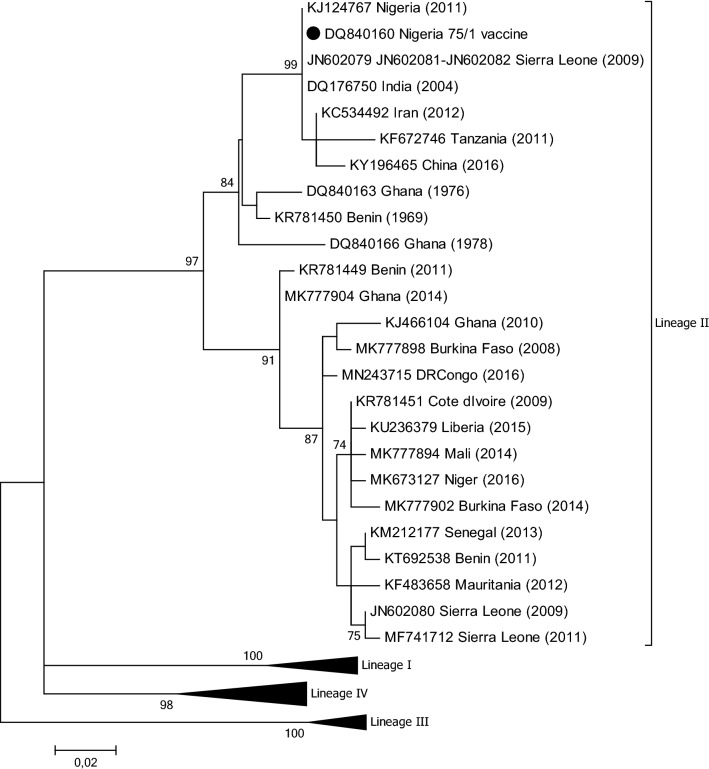
Phylogenetic tree based on partial sequences of the N protein (217 bp) gene of representative PPRV isolates of lineage II, indicating sequences that are similar to the vaccine strain Nigeria 75/1, indicated by a filled black circle. The tree was constructed using the maximum-likelihood (ML) method available in MEGA6, employing the Kimura 2-parameter model of nucleotide substitution and 500 bootstrap replications. The model was selected by MEGA6 as the best for the sequences being analysed

The analysis of 33 samples collected during outbreaks in 2007 and 2009 in two states (Kaduna and Plateau) in Nigeria identified lineage II viruses (assigned to lineage I by the authors according to the classification used at the time) [9]. A second molecular epidemiological study of 140 clinical samples from sheep and goats collected in Nigeria between 2010 and 2013 revealed that viruses from lineages II and IV were circulating in the country and that the lineage IV isolates grouped into two clades (IV-NigA and IV-NigB) [17] (Fig. 1). One of these viral sequences (GenBank no. KJ124767) was identical to the vaccine strain Nigeria 75/1 (Fig. 4 and Fig. 6), and this is most likely due to laboratory contamination during the processing of this sample.

Mantip et al. [77] confirmed the presence of both lineage II and lineage IV PPRV in Nigeria in a 2016 publication.

## Republic of Congo

There are no publications describing the presence of PPR in the Republic of Congo, although four outbreaks resulting in the death of 399 animals were reported to the OIE in 2006 [78].

## Rwanda

There is no information on the presence of PPR in Rwanda available in the literature, and no reports have been submitted to the OIE.

## Sahrawi territories

De Nardi et al. [79] investigated outbreaks in the Sahrawi Territories in 2010, and although it was claimed by the authors that the causative agent was a lineage IV PPRV with high nucleotide sequence similarity to Moroccan isolates, no sequence data are available in the public databases [79].

## Senegal

The earliest report of PPR in Senegal dates back to 1956 [52]. There is another report of a PPR outbreak (of unknown date) in West African dwarf and West African long-legged goats housed at the Institut Senegalais de Recherche Agricoles (ISRA) Dakar, Senegal, that has been associated with a lineage I virus (GenBank no. DQ840174) that was isolated in 1994 [52, 80] (Fig. 5). In 2013 a lyophilized specimen was shipped by ISRA to AGES for further characterization by the APHL. The specimen dated back to 1969, and although the original sample from which the specimen was derived is believed to have been collected in Senegal, the sample’s exact origin is unclear. The sample, however, had been used in animal experimentation in which goats had been infected and had shown clear clinical signs of PPR. The full genome of this virus was sequenced (GenBank no. KP789375), and phylogenetic analysis showed that the virus belonged to lineage I (Fig. 5). A second full genome of a lineage II virus from Senegal has also been sequenced (GenBank no. KM212177) [81] (Fig. 4).

## Sierra Leone

In 2009, blood samples from suspected outbreaks of PPR in central Sierra Leone were collected. Phylogenetic analysis of partial N gene sequences amplified from PPRV RNA in the samples identified one virus (GenBank no. JN602080) that clustered with PPRV isolates from Mali, whereas all of the others (GenBank nos. JN602079, JN602081 and JN602082) showed 100% sequence identity with the vaccine strain Nigeria 75/1 [82] (Fig. 4). The authors concluded that, since PPR vaccination was not being performed at the time in Sierra Leone, the sequences obtained were from circulating field viruses that were related to Nigeria 75/1 rather than being vaccine-derived. However, this seems very unlikely and was most probably due to either laboratory contamination or the unreported use of PPR vaccination in the country. More recently, a full genome sequence of PPRV RNA extracted from samples collected in 2011 has been determined (GenBank no. MF741712). Specifically, in December 2011, in Moyamba, south-western Sierra Leone, pathological and swab samples were collected from goats during a suspected PPR outbreak. Phylogenetic analysis of the amplicons generated from positive tissue samples revealed that they contained viral RNA from a lineage II PPRV that was not similar to the Nigeria 75/1 vaccine strain [83] (Fig. 4).

## Somalia

Authorities in Somalia reported the presence of PPR in the country to the OIE in 2009 [84]. Other than this, there are no studies in the literature describing PPR in Somalia.

## Sudan

Two viruses collected in central Sudan in 1971 and 1972 following outbreaks in goats were initially believed to be rinderpest virus but were subsequently identified as PPRV [85]. There are two sequence submissions in the GenBank database (nos. DQ840158 and FN996000) of PPRV isolates from 1972 that show that the virus belongs to lineage III (Fig. 3). Several serological studies were undertaken from 2000 to 2010 [86–88] confirming the presence of PPRV in Sudan. In 2011 it was reported by Kwiatek et al. [13] that the PPRV strains circulating in in the country belonged to lineage III and lineage IV (Figs. 2 and 3). Of the 64 PPRV-positive samples collected from 2000 to 2009 that were sequenced by Kwiatek et al. [13], only two belonged to lineage III, leading the authors to conclude that this lineage was being progressively replaced by lineage IV viruses, a situation that has also been reported recently in Ethiopia [49].

In May 2017, PPR was reported in free-ranging dorcas gazelles in Dinder National Park, south-eastern Sudan, with clinical signs of disease [89]. Interestingly, PPRV was also detected in healthy semi-captive gazelles. Viruses in both cases were further characterized and shown to belong to lineage IV. This study is of interest, as it identified a potential wildlife reservoir of PPRV in Africa.

## South Sudan

In 2011, 18 samples were received from South Sudan by APHL following suspected outbreaks in the country. Viruses were isolated and characterized and shown to belong to lineage IV (GenBank nos. MT543151- MT543152) (Fig. 2).

## Tanzania

In 2008, a serological survey of sheep and goats carried out in northern Tanzania reported an overall seropositivity of 45.8% [90]; this was the first report of PPR in Tanzania. Several other serological studies have been performed confirming the circulation of PPRV in the country, but viral characterization was not undertaken and reported until 2014 [91]. Analysis of samples collected from goats in northern and eastern regions of Tanzania identified the presence of lineage III viruses (Fig. 3) [91]. This was confirmed by Jones et al. in a very recent publication [92]. There are two reports that describe the identification of lineage II PPRV in neighbouring Tanzania. A partial N gene sequence (GenBank no. KF672746) from a lineage II PPRV was identified in a goat in southern Tanzania in 2011 [93]. In addition, there is a report of lineage II PPRVs identified in sheep in northern Tanzania in 2014, although the sequences are presently not publicly available [94]. An analysis of sequence KF672746, however, shows that it is very similar to the Nigeria 75/1 vaccine strain (see Fig. 4). Likewise, the phylogenetic tree presented by Mahaptra et al. [94] indicates a high similarity between their sequences and sequence KF672746. This suggests that there may be a PPRV circulating inTanzania that is very similar to, or even a variant of, the vaccine strain Nigeria 75/1 or that the results from these studies are due to laboratory contamination. In the study by Misinzo et al. [93], the authors also identified a lineage IV virus in the southern part of Tanzania. However, the sequence of this virus (GenBank no. KK672745) is also of note, as it is more similar to lineage IV viruses from Iran and Turkey than to viruses from Africa. Therefore, whether lineage II or lineage IV viruses are actually circulating in Tanzania needs to be confirmed..

## Togo

The first report of PPR inTogo was in 1972 [95]. In 2005, the death of 1560 animals from 90 PPR outbreaks was reported to the OIE by Togolese authorities [96]. No reports on the characterization of PPRV in Togo have been published.

## Tunisia

The first serological study for PPR was carried out on samples collected between September 2006 and January 2007 in Tunisia, and the seroprevalence was found to be 7.45% [97]. Between September 2012 and January 2013, clinical signs compatible with PPR in flocks of sheep and goats were reported to the Tunisian veterinary service, and samples were tested for PPRV. Viral samples from two separate outbreaks were sequenced, and phylogenetic analysis revealed that they both belonged to lineage IV, similar to other North African PPRV isolates [98] (Fig. 2). A further 86 outbreaks were reported in  Tunisia in 2016, and molecular characterization of the strains involved confirmed the circulation of lineage IV viruses in the country [99]. However, the PPRV isolates identified in 2016  were more similar to viruses from Egypt than to those from Morocco or Algeria, which was the case for PPRVs identified in Tunisia in 2012 (Fig. 2).

## Uganda

Antibodies against PPRV were first reported in small ruminants in eastern African countries, including Uganda, in 1995 [54], while the first molecular epidemiological characterization was published in 2012 [100]. Luka et al. erroneously described the presence of lineages I, II and IV in Uganda following the partial sequencing of the F gene from six viral samples [100]. A reanalysis of these sequences (GenBank nos. HQ407497 to HQ407502) indicates that the actual lineages present in Uganda were lineages IV and III. However, the lineage IV sequences were very similar to viruses from India and Nigeria, suggesting a possible laboratory contamination (Fig. S1). The full genome sequence of a lineage III virus from Uganda was determined in 2014 [46]. In 2019, Nkamwesiga et al. identified further lineage III PPRV isolates in Uganda and, interestingly, showed that there were two distinct clusters associated with the northern and southern part of the country that were more closely related to other East African isolates than to each other (Fig. 3) [101].

## Lineages in Africa

All four PPRV lineages are present in Africa, as shown in Fig. 1. The identification of lineage I viruses is confined to four West African countries (Côte d’Ivoire, Guinea Bissau, Guinea, and Niger). It now appears that lineage I viruses are no longer circulating, as this lineage has not been identified since 2001 [69]. Some argue that this lineage has been replaced by lineage II and IV viruses across the region [49, 81]. Lineage II is predominantly present in West Africa, although it has recently been reported in the DRC and Tanzania. As discussed above, the presence of circulating lineage II viruses in Tanzania needs to be confirmed. Lineage III has been reported in the Comoros islands and in northeastern, eastern, and central Africa but has not been seen in the north or west of the continent. Having been reported in fifteen countries, lineage IV is the predominant lineage seen in Africa. To date, it has been identified in the northern, western, central and eastern regions Africa and is gradually moving southwards.

From the phylogenetic trees presented in Figs. 2, 3, 4 and 5 it can be seen that there are some noteworthy differences between the viruses included in the analyses. As would be expected, there is evident nucleotide sequence divergence over time between the lineage I viruses identified in 1968–1969 in Senegal and others collected 20 to 40 years later in Burkina Faso, Côte d’Ivoire, Guinea Bissau and Niger (i.e., 1988, 1989 and 2011) (Fig. 5). Similarly, for lineage II viruses there are two distinct clusters visible: one consisting of sequences from viruses identified from 1969 to 1978, including the Nigerian vaccine strain and those from 1999 to 2016 (Fig. 4). There are differences between earlier lineage III viruses from 1972 and 1994 and those from 2011 to 2018 (Fig. 3). For lineage IV there appear to be geographical differences between the viruses identified in the north and east of Africa and west, central and southern Africa (Fig. 2). However, not all of the viruses fall into these groupings: the exceptions are PPRV isolates identified in Cameroon in 1997, Sudan in 2000, Central African Republic in 2004, Eritrea in 2002 to 2005, South Sudan in 2011, and Tanzania in 2011. Nevertheless, with the increasing dominance of lineage IV in Africa, as seen already in Asia and the Middle East, the identification of subgroups may make it easier to understand and monitor the spread of this virus through the continent.

## Conclusions

There is a very limited amount of molecular epidemiological data available on PPRV in wildlife at the global level. For Africa there are just a few serological studies that describe seropositivity for PPRV in buffaloes, gazelle, hartebeests, impala, kobs, waterbucks and wildebeest [94, 102]. No genetic characterization of the virus in the wild ruminants was performed. Recently, and as mentioned previously, a study by Asil et al. [89] identified lineage IV PPRV in dorcas gazelles in Sudan. Since wildlife can be significantly threatened themselves by the disease [103–105], the characterization of PPRV isolates collected from wild ruminants should be encouraged, and resources to enable the collection and analysis of samples should be identified.

This review has highlighted publications on PPRV in Africa that have identified lineage II viruses from Nigeria, Sierra Leone, and Tanzania that are either identical or very similar to the Nigeria 75/1 vaccine [17, 82, 93]. This issue is not confined to Africa, as can been seen by a search of the public databases, which reveals a number of PPRV sequences supposedly from field viruses that have a high degree of nucleotide sequence similarity to Nigeria 75/1. These include viruses from China (GenBank no. KY196465) [106], India (GenBank no. DQ176750) and Iran (GenBank no. KC534492) (Fig. 6). In agreement with Liu et al. [107], we believe that the majority of these cases are most likely  due to laboratory contamination during the handling of samples.

It is evident from this review that not all of the PPRV sequence data generated to date have been submitted to public databases. This prevents the timely sharing and evaluation of important data, which is essential for a better understanding of virus movement and spread. Laboratories that have sample collections with both historical and recent samples should characterize, release, and share information as quickly as possible.

Why lineage IV has become the predominant lineage globally and is now making its way through Africa is unclear. It has not been experimentally determined whether the virus is more or less pathogenic than viruses of other lineages, whether it is more or less susceptible to vaccines, whether it is transmitted more easily, or whether it is more stable in the environment. Similarly, why lineage I viruses appear to have disappeared is an intriguing question that awaits an explanation.

Finally, updates, confirmation, and characterization of PPR in Chad, Djibouti, Guinea, Guinea-Bissau, Libya, Rwanda, Somalia, and Togo are required. Data regarding the status of PPR in all African countries are essential if the eradication of this devastating disease is to be successful.

## Electronic supplementary material

Below is the link to the electronic supplementary material.

Supplementary file1 Fig. S1 Phylogenetic tree based on partial sequences of the F protein (167 bp) gene of representative PPRV isolates of lineage III and lineage IV. The tree was constructed using the maximum-likelihood (ML) method available in MEGA6, employing the Kimura 2-parameter model of nucleotide substitution and 500 bootstrap replications. The model was selected by MEGA6 as the best for the sequences being analysed. Samples from Uganda are indicated by filled black circles. (DOCX 23 kb)

## References

[1] Amarasinghe GK, Bào Y, Basler CF, Bavari S, Beer M, Bejerman N, Blasdell KR, Bochnowski A, Briese T, Bukreyev A, Calisher CH, Chandran K, Collins PL, Dietzgen RG, Dolnik O, Dürrwald R, Dye JM, Easton AJ, Ebihara H, Fang Q, Formenty P, Fouchier RAM, Ghedin E, Harding RM, Hewson R, Higgins CM, Hong J, Horie M, James AP, Jiāng D, Kobinger GP, Kondo H, Kurath G, Lamb RA, Lee B, Leroy EM, Li M, Maisner A, Mühlberger E, Netesov SV, Nowotny N, Patterson JL, Payne SL, Paweska JT, Pearson MN, Randall RE, Revill PA, Rima BK, Rota P, Rubbenstroth D, Schwemmle M, Smither SJ, Song Q, Stone DM, Takada A, Terregino C, Tesh RB, Tomonaga K, Tordo N, Towner JS, Vasilakis N, Volchkov VE, Wahl-Jensen V, Walker PJ, Wang B, Wang D, Wang F, Wang LF, Werren JH, Whitfield AE, Yan Z, Ye G, Kuhn JH (2017). Taxonomy of the order Mononegavirales: update. Arch Virol.

[2] Bailey D, Banyard A, Dash P, Ozkul A, Barrett T (2005). Full genome sequence of peste des petits ruminants virus, a member of the Morbillivirus genus. Virus Res.

[3] Rima B, Balkema-Buschmann A, Dundon WG, Duprex P, Easton A, Fouchier R, Kurath G, Lamb R, Lee B, Rota P, Wang L (2019). ICTV report Consortium ICTV virus taxonomy profile: *Paramyxoviridae*. J Gen Virol.

[4] Diallo A, Barrett T, Barbron M, Meyer G, Lefèvre PC (1994). Cloning of the nucleocapsid protein gene of peste-des-petits-ruminants virus: relationship to other morbilliviruses. J Gen Virol.

[5] Meyer G, Diallo A (1995). The nucleotide sequence of the fusion protein gene of the peste des petits ruminants virus: the long untranslated region in the 5'-end of the F-protein gene of morbilliviruses seems to be specific to each virus. Virus Res.

[6] Couacy-Hymann E, Roger F, Hurard C, Guillou JP, Libeau G, Diallo A (2002). Rapid and sensitive detection of peste des petits ruminants virus by a polymerase chain reaction assay. J Virol Methods.

[7] Kwiatek O, Keita D, Gil P, Fernández-Pinero J, Jimenez Clavero MA, Albina E, Libeau G (2010). Quantitative one-step real-time RT-PCR for the fast detection of the four genotypes of PPRV. J Virol Methods.

[8] Abubakar M, Rajput ZI, Arshed MJ, Sarwar G, Ali Q (2011). Evidence of peste des petits ruminants virus (PPRV) infection in Sindh Ibex (Capra aegagrus blythi) in Pakistan as confirmed by detection of antigen and antibody. Trop Anim Health Prod.

[9] Luka PD, Erume J, Mwiine FN, Ayebazibwe C, Shamaki D (2011). Molecular characterization and phylogenetic study of peste des petits ruminants viruses from north central states of Nigeria. BMC Vet Res.

[10] Munir M, Saeed A, Abubakar M, Kanwal S, Berg M (2015). Molecular characterization of peste des petits ruminants viruses from outbreaks caused by unrestricted movements of small ruminants in Pakistan. Transbound Emerg Dis.

[11] Shaila MS, Shamaki D, Forsyth MA, Diallo A, Goatley L, Kitching RP, Barrett T (1996). Geographic distribution and epidemiology of peste des petits ruminants virus. Virus Res.

[12] Banyard AC, Parida S, Batten C, Oura C, Kwiatek O, Libeau G (2010). Global distribution of peste des petits ruminants virus and prospects for improved diagnosis and control. J Gen Virol.

[13] Kwiatek O, Ali YH, Saeed IK, Khalafalla AI, Mohamed OI, Obeida AA, Abdelrahman MB, Osman HM, Taha KM, Abbas Z, El Harrak M, Lhor Y, Diallo A, Lancelot R, Albina E, Libeau G (2011). Asian lineage of peste des petits ruminants virus, Africa. Emerg Infect Dis.

[14] Kumar KS, Babu A, Sundarapandian G, Roy P, Thangavelu A, Kumar KS, Arumugam R, Chandran ND, Muniraju M, Mahapatra M, Banyard AC, Manohar BM, Parida S (2014). Molecular characterisation of lineage IV peste des petits ruminants virus using multi gene sequence data. Vet Microbiol.

[15] Cosseddu GM, Pinoni C, Polci A, Sebhatu T, Lelli R, Monaco F (2013). Characterization of peste des petits ruminants virus, Eritrea, 2002–2011. Emerg Infect Dis.

[16] Maganga GD, Verrier D, Zerbinati RM, Drosten C, Drexler JF, Leroy EM (2013). Molecular typing of PPRV strains detected during an outbreak in sheep and goats in south-eastern Gabon in 2011. Virol J.

[17] Woma TY, Adombi CM, Yu D, Qasim AM, Sabi AA, Maurice NA, Olaiya OD, Loitsch A, Bailey D, Shamaki D, Dundon WG, Quan M (2016). Co-circulation of peste-des-petits-ruminants virus Asian lineage IV with lineage II in Nigeria. Transbound Emerg Dis.

[18] FAOSTAT Food and Agriculture Data of the Food and Agriculture Organization of the United Nations. (https://www.fao.org/faostat/en/#data/QA) Accessed June 2020

[19] Global Strategy for the Control and Eradication of PPR (2015). (https://www.fao.org/3/a-i4460e.pdf) Accessed June 2020

[20] Saravanan P, Sen A, Balamurugan V, Rajak KK, Bhanuprakash V, Palaniswami KS, Nachimuthu K, Thangavelu A, Dhinakarraj G, Hegde R, Singh RK (2010). Comparative efficacy of peste des petits ruminants (PPR) vaccines. Biologicals.

[21] Kumar N, Barua S, Riyesh T, Tripathi BN (2017). Advances in peste des petits ruminants vaccines. Vet Microbiol.

[22] Mariner JC, Gachanja J, Tindih SH, Toye P (2017). A thermostable presentation of the live, attenuated peste des petits ruminants vaccine in use in Africa and Asia. Vaccine.

[23] Kardjadj M, Ben-Mahdi MH, Luka PD (2015). First serological and molecular evidence of PPRV occurrence in Ghardaïa district, center of Algeria. Trop Anim Health Prod.

[24] Baazizi R, Mahapatra M, Clarke BD, Ait-Oudhia K, Khelef D, Parida S (2017). Peste des petits ruminants (PPR): a neglected tropical disease in Maghreb region of North Africa and its threat to Europe. PLoS ONE.

[25] OIE (2012) WAHIS Country report, Angola. https://www.oie.int/wahis_2/public/wahid.php/Reviewreport/Review?page_refer=MapFullEventReport&reportid=12408. Accessed June 2020

[26] Niyokwishimira A, de D Baziki J, Dundon WG, Nwankpa N, Njoroge C, Boussini H, Wamwayi H, Jaw B, Cattoli G, Nkundwanayo C, Ntakirutimana D, Balikowa D, Nyabongo L, Zhang Z, Bodjo SC (2019). Detection and molecular characterization of Peste des Petits Ruminants virus from outbreaks in Burundi, December 2017-January 2018. Transbound Emerg Dis.

[27] Mornet P, Orue J, Gillbert Y, Thiery G, Mamadou S (1956). La peste des petits ruminants en Afrique occidental francaise; ses rapports avec la peste bovines. Rev Elev Med Vet Pays Trop.

[28] OIE (1996) Handistatus II Country report, Benin. https://web.oie.int/hs2/sit_pays_mald_pl.asp?c_pays=15&c_mald=6. Accessed June 2020

[29] Adombi CM, Waqas A, Dundon WG, Li S, Daojin Y, Kakpo L, Aplogan GL, Diop M, Lo MM, Silber R, Loitsch A, Diallo A (2017). Peste des petits ruminants in Benin: persistence of a single virus genotype in the country for over 42 years. Transbound Emerg Dis.

[30] Tounkara K, Wiatek O, Niang M, Sidibe CAK, Sery A, Dakouo M, Salami H, Lo MM, Ba A, Diop M, El Mamy AB, El Arbi AS, Barry Y, Isselmou E, Habiboullah H, Lella AS, Doumbia B, Gueya MB, Svadogo J, Ouattara L, Minougou G, Libeau G, Bataille A (2019). Genetic evidence for transboundary circulation of peste des petits ruminants across West Africa. Front Vet Sci.

[31] Ndamukong KJ, Sewell MM, Asanji MF (1989). Disease and mortality in small ruminants in the North West Province of Cameroon. Trop Anim Health Prod.

[32] Awa DN, Njoya A, Ngo Tama AC (2000). Economics of prophylaxis against peste des petits ruminants and gastrointestinal helminthosis in small ruminants in north Cameroon. Trop Anim Health Prod.

[33] Bidjeh K, Bornarel P, Imadine M, Lancelot R (1995). First- time isolation of the peste des petits ruminants (PPR) virus in Chad and experimental induction of the disease. Rev Elev Med Vet Pays Trop.

[34] Mahamat O, Doungous T, Kebkiba B, Oumar HA, Oussiguéré A, Yacoub AH, Goudja A, Guindé M, Moussa AH (2018). Seroprevalence, geographical distribution, and risk factors of peste des petits ruminants in the Republic of Chad. J Adv Vet Anim Res.

[35] Cêtre-Sossah C, Kwiatek O, Faharoudine A, Soulé M, Moutroifi YO, Vrel MA, Salami H, Rassoul S, Asnaoui M, Moindjie Y, Albina E, Libeau G, Cardinale E (2016). Impact and epidemiological investigations into the incursion and spread of Peste des Petits Ruminants in the Comoros Archipelago: an increased threat to surrounding islands. Transbound Emerg Dis.

[36] Gargadennec L, Lalanne A (1942). Peste des petits ruminants. Bull Services Zootechniques Epizzoties l’Afrique Occidentale Francaise.

[37] Chard LS, Bailey DS, Dash P, Banyard AC, Barrett T (2008). Full genome sequences of two virulent strains of peste-des-petits ruminants virus, the Côte d'Ivoire 1989 and Nigeria 1976 strains. Virus Res.

[38] OIE (2012) WAHIS Country report, DRC. https://www.oie.int/wahis_2/public/wahid.php/Reviewreport/Review?page_refer=MapFullEventReport&reportid=11810. Accessed June 2020

[39] Tshilenge GM, Walandila JS, Kikukama DB, Masumu J, Katshay Balowa L, Cattoli G, Bushu E, Mpiana Tshipambe S, Dundon WG (2019). Peste des petits ruminants viruses of lineages II and III identified in the Democratic Republic of the Congo. Vet Microbiol.

[40] Teshale S, Moumin G, Moussa C, Gezahegne M (2018). Seroprevalence and risk factors for peste des petits ruminants in sheep and goats in Djibouti. Rev Sci Tech.

[41] Ismail IM, House J (1990). Evidence of identification of peste des petits ruminants from goats in Egypt. Arch Exp Veterinarmed.

[42] Moustafa T (1993). Rinderpest and peste des petits ruminants-like disease in the Al-Ain region of the United Arab Emirates. Rev Sci Tech.

[43] Abd El-Rahim IH, Sharawi SS, Barakat MR, El-Nahas EM (2010). An outbreak of peste des petits ruminants in migratory flocks of sheep and goats in Egypt in 2006. Rev Sci Tech.

[44] Soltan MA, Abd-Eldaim MM (2014). Emergence of peste des petits ruminants virus lineage IV in Ismailia Province. Egypt Infect Genet Evol.

[45] Roeder PL, Abraham G, Kenfe G, Barrett T (1994). Peste des petits ruminants in Ethiopian goats. Trop Anim Health Prod.

[46] Muniraju M, Munir M, Banyard AC, Ayebazibwe C, Wensman J, Zohari S, Berg M, Parthiban AR, Mahapatra M, Libeau G, Batten C, Parida S (2014). Complete Genome Sequences of Lineage III Peste des Petits Ruminants Viruses from the Middle East and East Africa. Genome Announc.

[47] Muniraju M, Mahapatra M, Ayelet G, Babu A, Olivier G, Munir M, Libeau G, Batten C, Banyard AC, Parida S (2016). Emergence of lineage IV peste des petits ruminants virus in Ethiopia: complete genome sequence of an Ethiopian isolate 2010. Transbound Emerg Dis.

[48] Alemu B, Gari G, Libeau G, Kwiatek O, Kidane M, Belayneh R, Siraw B, Wieland B, Asfaw W, Abdi RD (2019). Molecular detection and phylogenetic analysis of Peste des petits ruminants virus circulating in small ruminants in eastern Amhara region. Ethiopia BMC Vet Res.

[49] Rume VN, Dundon WG, Belay G, Baziki JD, Diakite A, Paul A, Tessema YD, Nwankpa N, Gizaw D, Cattoli G, Bodjo SC, Tessema TS (2019). Molecular epidemiological update of peste des petits ruminants virus (PPRV) in Ethiopia. Vet Microbiol.

[50] Sumption KJ, Aradom G, Libeau G, Wilsmore AJ (1998). Detection of peste des petits ruminants virus antigen in conjunctival smears of goats by indirect immunofluorescence. Vet Rec.

[51] Anderson J, McKay JA (1994). The detection of antibodies against peste des petits ruminants virus in cattle, sheep and goats and the possible implications to rinderpest control programmes. Epidemiol Infect.

[52] Kwiatek O, Minet C, Grillet C, Hurard C, Carlsson E, Karimov B, Albina E, Diallo A, Libeau G (2007). Peste des petits ruminants (PPR) outbreak in Tajikistan. J Comp Pathol.

[53] Dundon WG, Adombi C, Waqas A, Otsyina HR, Arthur CT, Silber R, Loitsch A, Diallo A (2014). Full genome sequence of a peste des petits ruminants virus (PPRV) from Ghana. Virus Genes.

[54] Wamwayi HM, Rossiter PB, Kariuki DP, Wafula JS, Barrett T, Anderson J (1995). Peste des petits ruminants antibodies in east Africa. Vet Rec.

[55] OIE (2005) WAHIS Country report Kenya. https://www.oie.int/wahis_2/public/wahid.php/Reviewreport/Review?page_refer=MapFullEventReport&reportid=4526. Accessed June 2020

[56] Dundon WG, Kihu SM, Gitao GC, Bebora LC, John NM, Oyugi JO, Loitsch A, Diallo A (2017). Detection and genome analysis of a lineage III peste des petits ruminants virus in kenya in 2011. Transbound Emerg Dis.

[57] Boussini H, Chitsungo E, Bodjo SC, Diakite A, Nwankpa N, Elsawalhy A, Anderson JR, Diallo A, Dundon WG (2016). First report and characterization of peste des petits ruminants virus in Liberia, West Africa. Trop Anim Health Prod.

[58] Almeshay MD, Gusbi A, Eldaghayes I, Mansouri R, Bengoumi M, Dayhum AS (2017). An epidemiological study on peste des petits ruminants in tripoli region, Libya. Vet Ital.

[59] Dayhum A, Sharif M, Eldaghayes I, Kammon A, Calistri P, Danzetta ML, Di Sabatino D, Petrini A, Ferrari G, Grazioli S, Pezzoni G, Brocchi E (2018). Sero-prevalence and epidemiology of peste des petits ruminants in Libya. Transbound Emerg Dis.

[60] Tounkara K, Traoré A, Traoré AP, Sidibé S, Samake K, Diallo BO, Diallo A (1996). Epidémiologie de la peste des petits ruminants (PPR) et de la peste bovine au Mali: enquêtes sérologiques. Rev Elev Med Vet Pays Trop.

[61] Kamissoko B, Sidibé CAK, Niang M, Samake K, Traoré A, Diakité A, Sangare O, Diallo A, Libeau G (2013). Seroprevalence of peste des petits ruminants in sheep and goats in Mali. Rev Elev Med Vet Pays Trop.

[62] Le Jan C, Sow AD, Thiemoko C, François JL, Diouara A (1987). Pneumopathies enzootiques des petits ruminants en Mauritanie: situation d’ensemble et approche expérimentale. Rev Elev Med Vet Pays Trop.

[63] Lemrabott OM, Elmamy OB, Diarra I, Baba OM, Bastiaensen P, Bendali F, Diop B, Kock R, Tounkara K, Bidjeh K, Thomson G, Fall M (2005). Peste bovine: limites de la sérologie? Cas de la Mauritanie. Rev Elev Med Vet Pays Trop.

[64] El Arbi AS, El Mamy AB, Salami H, Isselmou E, Kwiatek O, Libeau G, Kane Y, Lancelot R (2014). Peste des petits ruminants virus, Mauritania. Emerg Infect Dis.

[65] Muniraju M, El Harrak M, Bao J, Ramasamy Parthiban AB, Banyard AC, Batten C, Parida S (2013). Complete genome sequence of a peste des petits ruminants virus recovered from an alpine goat during an outbreak in Morocco in 2008. Genome Announc.

[66] Fakri F, Embarki T, Parida S, Bamouh Z, Jazouli M, Mahapatra M, Tadlaoui K, Fassi-Fihri O, Richardson CD, Elharrak M (2016). Re-emergence of Peste des Petits Ruminants virus in 2015 in Morocco: Molecular characterization and experimental infection in Alpine goats. Vet Microbiol.

[67] Bloch N, Diallo I (1991). Serological survey of small ruminants in 4 districts of Niger. Rev Elev Med Vet Pays Trop.

[68] Farougou S, Gagara M, Mensah GA (2013). Prevalence of peste des petits ruminants in the arid zone in the Republic of Niger. Onderstepoort J Vet Res.

[69] Tounkara K, Bataille A, Adombi CM, Maikano I, Djibo G, Settypalli TBK, Loitsch A, Diallo A, Libeau G (2018). First genetic characterization of peste des petits ruminants from Niger: on the advancing front of the Asian virus lineage. Transbound Emerg Dis.

[70] Souley MM, Issa Ibrahim A, Sidikou D, Dundon WG, Cattoli G, Abdou A, Soumana F, Yaou B (2019). Molecular epidemiology of peste des petits ruminants in Niger: an update. Transbound Emerg Dis.

[71] Gibbs EP, Taylor WP, Lawman MJ (1977). The isolation of adenoviruses from goats affected with peste des petits ruminants in Nigeria. Res Vet Sci.

[72] Obi TU, Ojo MO, Durojaiye OA, Kasali OB, Akpavie S, Opasina DB (1983). Peste des petits ruminants (PPR) in goats in Nigeria: clinical, microbiological and pathological features. Zentralbl Veterinarmed B.

[73] Obi TU, Rowe LW, Taylor WP (1984). Serological studies with peste des petits ruminants and rinderpest viruses in Nigeria. Trop Anim Health Prod.

[74] Opasina BA, Putt SNH (1985). Outbreaks of peste des petits ruminants in village goat flocks in Nigeria. Trop Anim Health Prod.

[75] Taylor WP, Abegunde A (1979). The isolation of peste des petits ruminants virus from Nigerian sheep and goats. Res Vet Sci.

[76] Diallo A, Taylor WP, Lefèvre PC, Provost A (1989). Attenuation of a strain of rinderpest virus: potential homologous live vaccine. Rev Elev Med Vet Pays Trop.

[77] Mantip S, Quan M, Shamaki D, Van Vuuren M (2016). Comparison of nucleotide sequences of recent and previous lineages of peste-des-petits-ruminants viruses of sheep and goats in Nigeria. Onderstepoort J Vet Res.

[78] OIE (2006) WAHIS Country report Republic of Congo. https://www.oie.int/wahis_2/public/wahid.php/Reviewreport/semestrial/review?year=2006&semester=1&wild=0&country=COG&this_country_code%E2%80%A6. Accessed June 2020

[79] De Nardi M, Lamin Saleh SM, Batten C, Oura C, Di Nardo A, Rossi D (2012). First evidence of peste des petits ruminants (PPR) virus circulation in Algeria (Sahrawi territories): outbreak investigation and virus lineage identification. Transbound Emerg Dis.

[80] Diop M, Sarr J, Libeau G (2005). Evaluation of novel diagnostic tools for peste des petits ruminants virus in naturally infected goat herds. Epidemiol Infect.

[81] Salami H, Croville G, Kwiatek O, Mariette J, Klopp C, Valière S, Guérin JL, Lo M, Thiongane Y, Albina E, Libeau G (2014). Complete genome sequence of a field strain of peste des petits ruminants virus isolated during 2010–2014 epidemics in Senegal. Genome Announc.

[82] Munir M, Zohari S, Suluku R, Leblanc N, Kanu S, Sankoh FA, Berg M, Barrie ML, Ståhl K (2012). Genetic characterization of peste des petits ruminants virus, Sierra Leone. Emerg Infect Dis.

[83] Dundon WG, Adombi CM, Kanu S, Loitsch A, Cattoli G, Diallo A (2018). Complete Genome Sequence of a lineage II peste des petits ruminants virus from Sierra Leone. Genome Announc.

[84] OIE (2009) WAHIS Country report Somalia. https://www.oie.int/wahis_2/public/wahid.php/Reviewreport/semestrial/review?year=2009&semester=1&wild=0&country=SOM&this_country_code%E2%80%A6. Accessed June 2020

[85] El Hag AB, Taylor WP (1984). Isolation of peste des petits ruminants virus from the Sudan. Res Vet Sci.

[86] Osman NA, Ali AS, A/Rahman ME, Fadol MA (2009). Antibody seroprevalences against Peste des Petits Ruminants (PPR) virus in sheep and goats in Sudan. Trop Anim Health Prod.

[87] Khalafalla AI, Saeed IK, Ali YH, Abdurrahman MB, Kwiatek O, Libeau G, Obeida AA, Abbas Z (2010). An outbreak of peste des petits ruminants (PPR) in camels in the Sudan. Acta Trop.

[88] Intisar KS, Ali YH, Haj MA, Sahar MA, Shaza MM, Baraa AM, Ishag OM, Nouri YM, Taha KM, Nada EM, Ahmed AM, Khalafalla AI, Libeau G, Diallo A (2017). Peste des petits ruminants infection in domestic ruminants in Sudan. Trop Anim Health Prod.

[89] Asil RM, Ludlow M, Ballal A, Alsarraj S, Ali WH, Mohamed BA, Mutwakil SM, Osman NA (2019). First detection and genetic characterization of peste des petits ruminants virus from dorcas gazelles "Gazella dorcas" in the Sudan, 2016–2017. Arch Virol.

[90] Swai ES, Kapaga A, Kivaria F, Tinuga D, Joshua G, Sanka P (2009). Prevalence and distribution of Peste des petits ruminants virus antibodies in various districts of Tanzania. Vet Res Commun.

[91] Kgotlele T, Macha ES, Kasanga CJ, Kusiluka LJ, Karimuribo ED, Van Doorsselaere J, Wensman JJ, Munir M, Misinzo G (2014). Partial genetic characterization of peste des petits ruminants virus from goats in northern and eastern Tanzania. Transbound Emerg Dis.

[92] Jones BA, Mahapatra M, Chubwa C, Clarke B, Batten C, Hicks H, Henstock M, Keyyu J, Kock R, Parida S (2020). Characterisation of Peste Des Petits Ruminants Disease in Pastoralist Flocks in Ngorongoro District of Northern Tanzania and Bluetongue Virus Co-Infection. Viruses.

[93] Misinzo G, Kgotlele T, Muse EA, Doorsselaere JV, Berg M, Munir M (2015). Peste des petits ruminants virus lineage II and IV From goats in southern Tanzania during an outbreak in 2011. British J Virol.

[94] Mahapatra M, Sayalel K, Muniraju M, Eblate E, Fyumagwa R, Shilinde L, Mdaki M, Keyyu J, Parida S, Kock R (2015). Spillover of peste des petits ruminants virus from domestic to wild ruminants in the serengeti ecosystem, Tanzania. Emerg Infect Dis.

[95] Benazet BGH (1973) La peste des petits ruminants: étude experimentale de la vaccination. Doctoral Thesis, University of Toulouse, France

[96] OIE (2005) WAHIS Country report Togo. https://www.oie.int/wahis_2/public/wahid.php/Reviewreport/semestrial/review?year=2005&semester=1&wild=0&country=TGO&this_country_code%E2%80%A6. Accessed June 2020

[97] Ayari-Fakhfakh E, Ghram A, Bouattour A, Larbi I, Gribâa-Dridi L, Kwiatek O, Bouloy M, Libeau G, Albina E, Cêtre-Sossah C (2011). First serological investigation of peste-des-petits-ruminants and Rift Valley fever in Tunisia. Vet J.

[98] Sghaier S, Cosseddu GM, Ben Hassen S, Hammami S, Ammar HH, Petrini A, Monaco F (2014). Peste des petits ruminants virus, Tunisia, 2012–2013. Emerg Infect Dis.

[99] Ben Hassen S, Monaco F, Sghaier S, Orsini M, Valleriani F, Haj Ammar H, Petrini A, Hammami S, Cosseddu GM (2018). Peste des Petits Ruminants outbreaks in Tunisia in 2016. Transbound Emerg Dis.

[100] Luka PD, Erume J, Mwiine FN, Ayebazibwe C (2012). Molecular characterization of peste des petits ruminants virus from the Karamoja region of Uganda (2007–2008). Arch Virol.

[101] Nkamwesiga J, Coffin-Schmitt J, Ochwo S, Mwiine FN, Palopoli A, Ndekezi C, Isingoma E, Nantima N, Nsamba P, Adiba R, Hendrickx S, Mariner JC (2019). Identification of peste des petits ruminants transmission hotspots in the Karamoja subregion of Uganda for targeting of eradication interventions. Front Vet Sci.

[102] Couacy-Hymann E, Bodjo C, Danho T, Libeau G, Diallo A (2005). Surveillance of wildlife as a tool for monitoring rinderpest and peste des petits ruminants in West Africa. Rev Sci Tech.

[103] Aguilar XF, Fine AE, Pruvot M, Njeumi F, Walzer C, Kock R, Shiilegdamba E (2018). PPR virus threatens wildlife conservation. Science.

[104] Kock RA, Orynbayev M, Robinson S, Zuther S, Singh NJ, Beauvais W, Morgan ER, Kerimbayev A, Khomenko S, Martineau HM, Rystaeva R, Omarova Z, Wolfs S, Hawotte F, Radoux J, Milner-Gulland EJ (2018). Saigas on the brink: Multidisciplinary analysis of the factors influencing mass mortality events. Sci Adv.

[105] Pruvot M, Fine AE, Hollinger C, Strindberg S, Damdinjav B, Buuveibaatar B, Chimeddorj B, Bayandonoi G, Khishgee B, Sandag B, Narmandakh J, Jargalsaikhan T, Bataa B, McAloose D, Shatar M, Basan G, Mahapatra M, Selvaraj M, Parida S, Njeumi F, Kock R, Shiilegdamba E (2020). Outbreak of Peste des Petits Ruminants among Critically Endangered Mongolian Saiga and Other Wild Ungulates, Mongolia, 2016–2017. Emerg Infect Dis.

[106] Zhou XY, Wang Y, Zhu J (2018). First report of peste des petits ruminants virus lineage II in Hydropotes inermis. China Transbound Emerg Dis.

[107] Liu F (2018). Letter to the editor concerning "First report of peste des petits ruminants virus lineage II in Hydropotes inermis, China" by Zhou et al. (Transbound Emerg Dis; 2017: https://doi.org/10.1111/tbed.12683). Transbound Emerg Dis.

